# The Diagnostic and Prognostic Value of miR-200c in Gastric Cancer: A Meta-Analysis

**DOI:** 10.1155/2019/8949618

**Published:** 2019-04-04

**Authors:** Zong-Sheng Huang, Xian-Wen Guo, Guo Zhang, Lie-Xin Liang, Bing Nong

**Affiliations:** Department of Gastroenterology, The People's Hospital of Guangxi Zhuang Autonomous Region, Nanning, China

## Abstract

**Background:**

The role of miR-200c in gastric cancer remains controversial. This study is aimed at clarifying the diagnostic and prognostic value of miR-200c in gastric cancer through a meta-analysis.

**Methods:**

A comprehensive literature search of PubMed, Embase, and Ovid library databases was conducted. The studies included were those conducted before December 2017. The sensitivity and specificity, positive likelihood ratio (PLR), negative likelihood ratio (NLR), diagnostic odds ratio (DOR), and area under curve (AUC) were used to estimate the diagnostic value of miR-200c. Meanwhile, the pooled hazard ratio (HR) was used to estimate the prognostic value of miR-200c.

**Results:**

For the diagnostic value of miR-200c, six studies that included 202 patients with gastric cancer and 250 normal controls were analyzed. The sensitivity, specificity, PLR, NLR, DOR, and AUC were 0.74, 0.66, 2.20, 0.40, 5.34, and 0.75, respectively. Subgroup analysis showed no significant difference in the type of the sample, method for testing miR-200c, and ethnicity among the patients. Meanwhile, for the prognostic value of miR-200c, seven studies comprising 935 patients with gastric cancer were analyzed. The pooled results showed that miR-200c expression was associated with overall survival (HR = 2.19) and disease-free survival (HR = 1.73), but not with progression-free survival (HR = 1.64) in patients with gastric cancer. There was no publication bias across the studies.

**Conclusions:**

Both serum and tissue miR-200c have moderate diagnostic accuracy in gastric cancer. miR-200c could also be used as a valuable indicator for predicting the prognosis of gastric cancer patients.

## 1. Background

Gastric cancer is among the most frequent types of cancer worldwide, particularly in Asian countries [1, 2]. Despite advances in surgery and other treatment modalities, the prognosis of patients with gastric cancer remains poor primarily because most patients are diagnosed at an advance stage [3]. Currently, the primary diagnostic modality for gastric cancer is gastroscopy, but this method is unsuitable as a first-line examination due to its invasive nature and the need for specially trained physicians. Moreover, there are no available biomarkers to predict the prognosis of patients with gastric cancer; thus, a reliable biomarker to diagnose gastric cancer and predict patient survival is needed, which will be beneficial to improve the clinical outcome of these patients.

miRNAs are endogenous small noncoding RNA molecules with a length of approximately 20–22 nucleotides. Studies have confirmed the relationship between specific cancers and many differential miRNAs, thus providing a new kind of disease-specific biomarkers for cancers [4]. As biomarkers, miRNAs can be used for cancer diagnosis and prediction of treatment outcomes and patient prognosis [5, 6]. miR-200c is a member of the miR-200 family and is associated with the development of several cancers, including gastric cancer [7]. In addition, miR-200c has been found to be an important diagnostic and prognostic indicator of some cancers, such as ovarian cancer [8], breast cancer [9], and pancreatic cancer [10].

The diagnostic and prognostic values of miR-200c in gastric cancer have also been reported in some studies; however, the results among these studies were inconsistent. In addition, the sample size of each study was small; thus, the conclusion drawn from the results was unreliable. As such, the diagnostic and prognostic values of miR-200c in gastric cancer remain uncertain. A meta-analysis is a quantitative method for combining data from multiple studies to achieve more reliable results compared with individual studies. This meta-analysis is aimed at clarifying the diagnostic and prognostic values of miR-200c in gastric cancer.

## 2. Materials and Methods

### 2.1. Literature Search

This study was conducted based on the guidelines proposed by the Human Genome Epidemiology Network for systematic review of genetic-association studies and complied with the PRISMA guidelines [11]. A comprehensive literature search of the Cochrane Clinical Trials Database, Medline (PubMed), Embase, Google Scholar, and Chinese National Knowledge Infrastructure database was conducted to identify studies that assessed the diagnostic or the prognostic value of miR-200c in gastric cancer. The included studies were those conducted before December 1st, 2017; the following keywords were used: “miR-200c,” “miRNA-200c,” “microRNA-200c,” “gastric cancer,” “stomach neoplasms,” and “stomach cancer.” The search was not limited by language or publication status. The search strategy of PubMed database was listed in Supplementary Materials (available here). The references of all retrieved publications were also reviews to check for additional relevant studies. Three blinded reviewers (HZS, GXW, and ZG) independently performed the literature search.

### 2.2. Inclusion and Exclusion Criteria

For studies evaluating the diagnostic value of miR-200c, the inclusion criteria were as follows: (1) gastric cancer was diagnosed via histopathology; (2) the study evaluated the diagnostic or prognostic value of miR-200c in gastric cancer; (3) the cut-off value of miR-200c was given, and sufficient data were provided to calculate the sensitivity and specificity. Meanwhile, for studies on the prognostic value of miR-200c, the inclusion criteria were as follows: (1) gastric cancer was diagnosed via histopathology; (2) the study evaluated the association of miR-200c expression with the prognosis of gastric cancer; (3) sufficient data were presented to calculate the HR and corresponding 95% CI. The exclusion criteria were as follows: laboratory studies, review articles, case reports, animal studies, or studies that did not provide sufficient data to calculate the diagnostic or prognostic value of miR-200c. If the same patient population was reported in several publications, the most recent study was selected for analysis.

### 2.3. Data Extraction and Quality Assessment

For the diagnostic value of miR-200c, the following data were extracted: (1) first author name, year of publication, country, and study design; (2) type of the sample; (3) number of patients with gastric cancer and controls; (4) stage of gastric cancer; (5) method for evaluating miR-200c; (6) miR-200c cut-off; and (7) sensitivity and specificity of miR-200c. For the prognostic value of miR-200c, the following data were extracted: (1) first author name, year of publication, country, and study design; (2) type of the sample; (3) number of patients with gastric cancer; (4) stage of gastric cancer; (5) method for evaluating miR-200c; (6) miR-200c cut-off; (7) outcome of prognosis; and (8) HR and corresponding 95% CI. The quality of individual studies was assessed using the quality assessment of diagnostic accuracy studies (QUADAS). QUADAS is an evidence-based quality assessment tool developed for use in systematic reviews; the highest possible score is 14, which indicates high quality of the study. Two blinded reviewers (HZS and GXW) independently extracted the data and assessed the quality of the studies. Disagreements between the reviewers were resolved through a discussion or by a third reviewer (ZG).

### 2.4. Statistical Analysis

For the analysis of diagnostic accuracy of miR-200c in gastric cancer, the summary diagnostic indexes, including sensitivity and specificity, positive likelihood ratio (PLR), negative likelihood ratio (NLR), and diagnostic odds ratio (DOR) with the corresponding 95% CIs, were calculated. PLR, NLR, and DOR were summarized using the random-effects model (DerSimonian-Laird method). The summary receiver operating characteristic (SROC) curve was applied to assess the overall diagnostic accuracy across the different threshold definitions. The results were described as the area under curve (AUC) of SROC with its *Q*∗-point representing the maximal joint sensitivity and specificity [12]. Subgroup analysis was carried out by dividing the studies according to different sample types, testing method, and ethnicity.

The pooled hazard ratio (HR) and its 95% CI were used to quantitatively determine the prognostic value of miR-200c in gastric cancer. Heterogeneity among studies was assessed using Cochran's *Q* test and the *I* ^2^ statistic. *I* ^2^ values <25%, 25%-50%, and >50% were set to indicate mild, moderate, and significant heterogeneity, respectively. Publication bias was assessed using Egger's test and Begg's test. Subgroup analysis was carried out by dividing the studies according to different sample types. All statistical tests in this meta-analysis were performed using Stata 11.2 software (StataCorp, College Station, TX) with two-tailed *P* values. A *P* value of <0.05 was considered statistically significant.

## 3. Results

### 3.1. Study Selection

**Figure 1:**
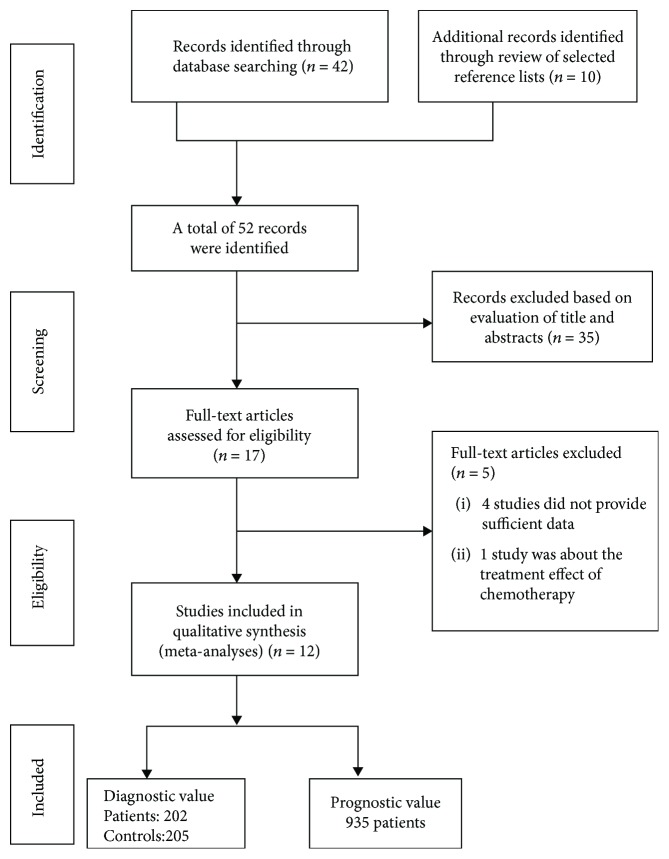
Flow chart of study selection.

The primary literature search from the databases retrieved 52 articles. After screening the titles and abstracts of the articles, 35 studies were excluded because they are irrelevant to the association of miRNAs with gastric cancer or were case reports, reviews, or animal studies. After reviewing the full text of the remaining 17 studies, five studies were further excluded because they did not provide sufficient data and they focused on the effect of chemotherapy [13]. The excluded studies were listed in Supplementary Materials. Finally, 12 studies [14–25] that included 1387 subjects were eligible for our meta-analysis. The flow chart of the study selection is shown in Figure 1.

### 3.2. Characteristics and Quality of the Included Studies

All cases of gastric cancer were diagnosed via histopathology. The patients from nine studies were Caucasians, while the patients in the other three studies were Asians. Gastric cancer was diagnosed using blood and tissue samples. miR-200c expression was assessed via quantitative reverse transcription polymerase chain reaction (RT-PCR) and microarray. The QUADAS scores ranged from 11 to 13. The characteristics of each included study and of the patients are described in detail in Tables 1 and 2.

### 3.3. Diagnostic Value of miR-200c in Gastric Cancer in Three Studies

Three studies used the microarray method to detect miRNA expression in gastric cancer tissue, but they did not provide the diagnostic value of miR-200c in gastric cancer. Therefore, we extracted the raw data to calculate the diagnostic value. In the study by Sierzega et al. [17], the sensitivity and specificity of miR-200c for diagnosing gastric cancer were 60.0% and 45.0%, respectively, at a cut-off of 0.3415. Meanwhile, in the study by Keller et al. [15], the sensitivity and specificity were 72.7% and 55.9%, respectively, at a cut-off value of 101.0392. In the study by Tseng et al. [20], the sensitivity and specificity were 54.5% and 54.5%, respectively, at a cut-off value of 2670.5.

### 3.4. Overall Analysis of the Diagnostic Value of miR-200c in Gastric Cancer

**Figure 2:**
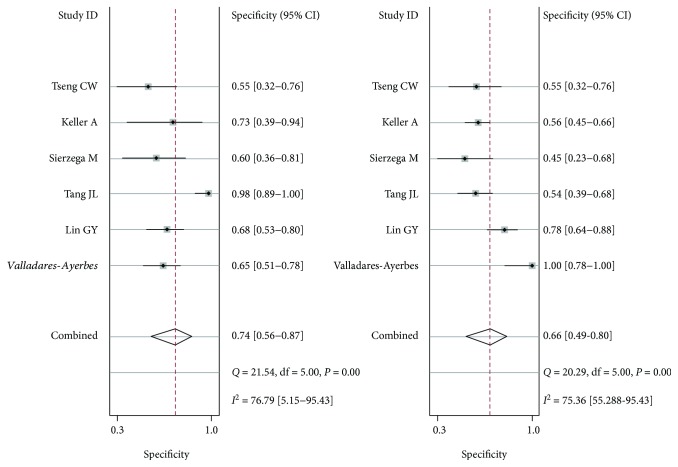
Sensitivity and specificity plotted graph for the diagnostic value of miR-200c in gastric cancer.

**Figure 3:**
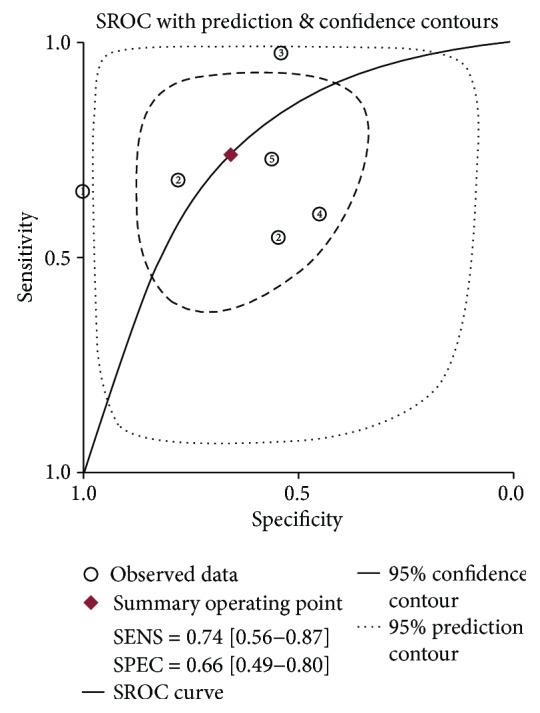
SROC curve plotted graph for the diagnostic value of miR-200c in gastric cancer.

**Figure 4:**
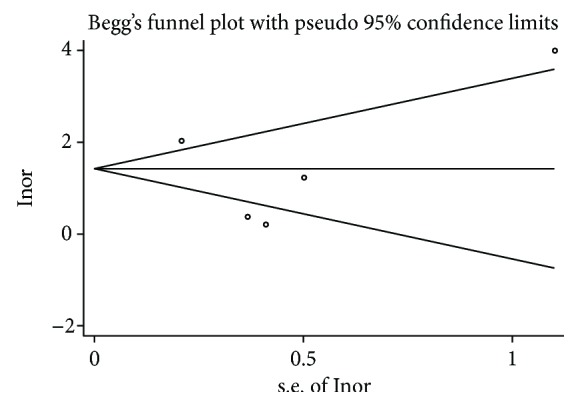
Publication bias plotted graph by Begg's test.

The summary estimates of the diagnostic indexes for miR-200c in gastric cancer are as follows: sensitivity, 0.74 (0.56-0.87); specificity, 0.66 (0.49-080); PLR, 2.20 (1.30-3.50); NLR, 0.40 (0.21-0.73); DOR, 5.34 (2.00-15); and AUC, 0.75 (0.71-0.79). The results had significant heterogeneity (*P* < 0.01) (Figures 2 and 3), but Egger tests (*P* = 0.759) and Begger's tests (*P* = 1.000) showed no evidence of significant publication bias (Figure 4).

### 3.5. Subgroup Analysis of the Diagnostic Value of miR-200c in Gastric Cancer

We next performed a subgroup analysis according to the type of the sample, testing method for miR-200c, and patient ethnicity. The results showed that the summary sensitivity and specificity were not significantly different according to the different types of the sample, testing method for miR-200c, and patient ethnicity (Table 3).

### 3.6. Overall Analysis of the Prognostic Value of miR-200c Expression in Gastric Cancer

**Figure 5:**
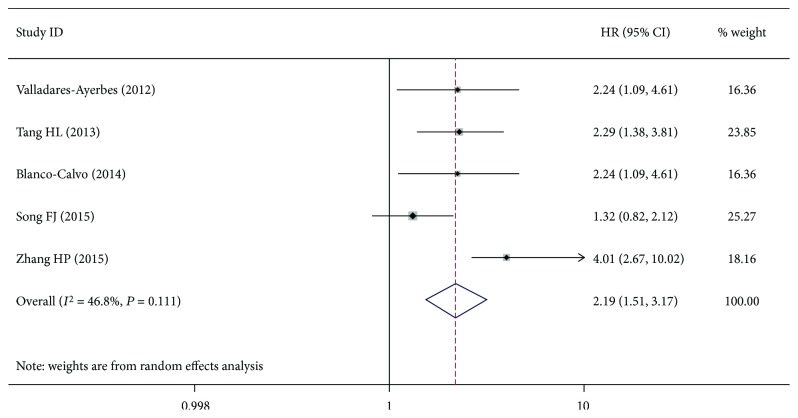
Meta-analysis of the miR-200a with OS in gastric cancer patients.

**Figure 6:**
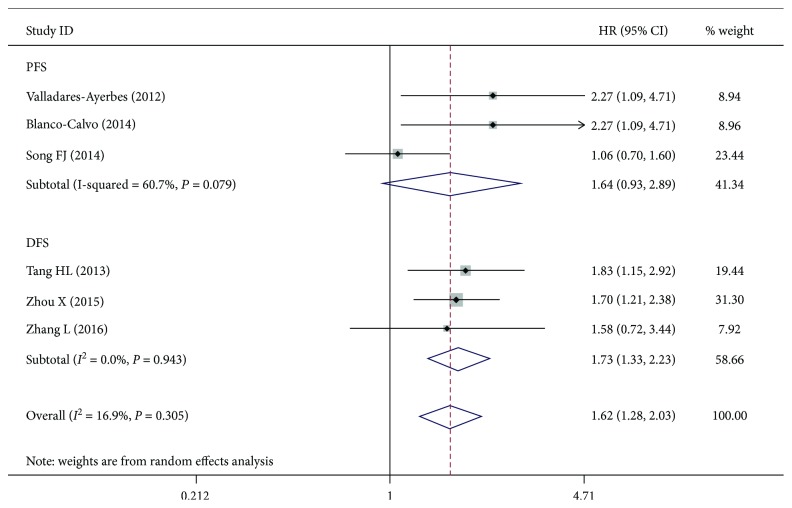
Meta-analysis of the miR-200a with PFS and DFS in gastric cancer patients.

**Figure 7:**
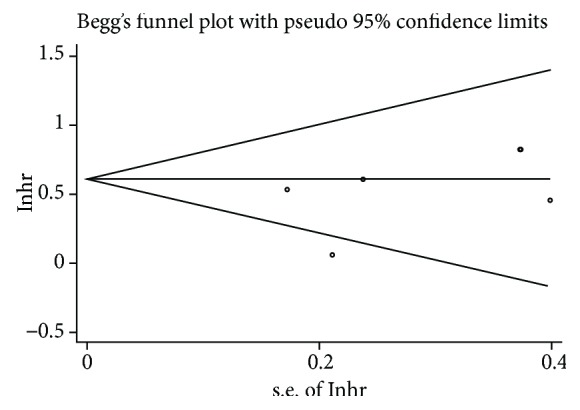
Publication bias plotted graph by Begg's test.

The pooled results of miR-200c expression was associated with overall survival (OS) in patients with gastric cancer (random-effects model: HR = 2.19, 95%CI = 1.851 − 3.17, *P* < 0.01), but with moderate heterogeneity (*I* ^2^ = 46.8%, *P* = 0.111; Figure 5). Egger and Begger's tests showed no evidence of significant publication bias (Egger's test = 0.100; Begger's test = 0.230). We also found that miR-200c was associated with disease-free survival (DFS) (HR = 1.73, 95%CI = 1.33 − 2.23, *P* < 0.01). No significant heterogeneity was found in the results. Meanwhile, miR-200c was not associated with progression-free survival (PFS) (HR = 1.64, 95%CI = 0.93 − 2.89, *P* = 0.086), and the results had no significant heterogeneity (*P* > 0.05) (Figure 6). No significant bias was also noted across the studies (*P* > 0.05) (Figure 7).

### 3.7. Sensitivity Analysis of the Prognostic Value of miR-200c Expression in Gastric Cancer

Only one study [18] used the tissue sample to investigate the association of miR-200c with OS. This study was excluded, and we found that the result remained similar to the overall results (HR = 2.194, 95%CI = 1.33 − 3.61). Regarding the association between miR-200c and DFS, one study [19] used the cut-off value but not the median expression value. We omitted this study and found that sensitivity result was in line with the overall results (HR = 1.68, 95%CI = 1.23 − 2.29).

## 4. Discussion

miRNAs have been found to be associated to various biological processes and to the development and progression of diseases [26]. Studies have shown that aberrant expression of miRNAs has the diagnostic and prognostic value in many kinds of cancers, including gastric cancer [27, 28]. Zheng et al. [28] evaluated the prognostic role of miRNAs in human gastrointestinal cancer using the meta-analysis method, and they found that several miRNAs, including miR-200c, were associated with the survival of gastrointestinal cancer. In the present study, we analyzed the diagnostic and prognostic value of miR-200c in gastric cancer through a meta-analysis. We found that miR-200c has moderate sensitivity and specificity in the diagnosis of gastric cancer, and the different sample types, testing method for miR-200c, or patient ethnicity did not influence the overall diagnostic accuracy. We also found that miR-200c was associated with the prognosis of gastric cancer, and patients with high expression of miR-200c have longer OS and DFS compared with those with low expression. Similar results were observed for PFS and DFS, suggesting that miR-200c could be used as a prognostic indicator for gastric cancer. Compared with Zheng et al.'s study [28], which only included two studies that investigated the prognostic value of miR-200c in gastric cancer, the present study included more articles, thus could greatly enhance the reliability of the results.

The role of miR-200c has been investigated in many cancers, and one study showed that miR-200c could promote or inhibit carcinogenesis depending on the type of cancers [29]. In gastric cancer, the role of miR-200c remains controversial as conflicting results have been obtained. Zhang et al. [23] reported that the expression of miR-200c was increased in the serum sample of gastric cancer, and high serum miR-200c level indicated a short survival time. Valladares-Ayerbes et al. [21] reported similar results. By contrast, Tang et al. [19] reported that miR-200c was downregulated in the tissue and cell sample of gastric cancer, and patients with high miR-200c expression have a long survival time. Similar results were obtained by Zhang et al. [23]. We analyzed the data reported by Keller et al. [15] and found contrasting results to those from the previous studies using tissue samples of gastric cancer. We found that miR-200c was upregulated in gastric cancer compared with normal controls. The above studies suggest that miR-200c plays a crucial role in the carcinogenesis of multiple types of cancers, including gastric cancer; however, whether it promotes or inhibits the development of gastric cancer remains unclear. We noted that there were differences regarding the association of ethnicities and sample types with gastric cancer pathogenesis and the diagnostic methods included RT-PCR and microarray. These factors might have influenced the results. In addition, the mechanism by which miR-200c affects the pathogenesis of gastric cancer needs to be further elucidated. Collectively, these conflicting results indicate the need for further studies on the role of miR-200c.

The diagnostic value of miR-200c in cancer has been reported in several studies. de Souza et al. [30] showed that the expression of serum miR-200c and miR-200b distinguished prostate cancer patients from controls, with a sensitivity and specificity of 67% and 75%, respectively. miR-200c also showed a high diagnostic accuracy for ovarian cancer, with a sensitivity and specificity of 84% and 83%, respectively [29]. Regarding gastric cancer, the diagnostic accuracy of miR-200c varied significantly, with a sensitivity ranging from 54.5% to 97.0% and a specificity ranging from 45.0% to 100%. Moreover, the sample size of each study, testing method for miR-200c, type of sample, and patient ethnicity were different. Therefore, we pooled the results of studies on the diagnostic value of miR-200c for gastric cancer and found that miR-200c has a moderate diagnostic accuracy regardless of the testing method for miR-200c, type of sample, and patient ethnicity. This result indicates that miR-200c can be used as a diagnostic indicator for gastric cancer.

For the prognostic value of miR-200c in gastric cancer, we found that a high expression of miR-200c was associated with a short survival time and patients with high miR-200c expression have poorer DFS than those with a low miR-200c expression. However, miR-200c expression was not associated with PFS. The sensitivity analysis further verified the robustness of the overall results. Our findings clarified the prognostic value of miR-200c in gastric cancer based on a large sample size. The results support that miR-200c has a high predictive value for the prognosis of patients with gastric cancer.

Previously, three meta-analysis studies [31–33] explored the prognostic role of miR-200 families in various cancers. Compared with these previous studies, the present study not only explored the prognostic role of miR-200c in gastric cancer but also explored the diagnostic role. In addition, the present study included more eligible studies compared with these previous studies, although we focus on the prognostic role of miR-200c instead of miR-200 families. Furthermore, we performed subgroup analysis based on the different clinical parameters, which were not reported in the previous meta-analysis. Therefore, the present meta-analysis provided more valuable information compared with the previous meta-analysis.

To our knowledge, this study was the first meta-analysis to clarify the diagnostic and prognostic value of miR-200c in gastric cancer. By combining all available data, our study was able to overcome the limitation of small sample size of individual studies and provide a more reliable estimation. Moreover, the absence of significant publication bias further supported the robustness of the results. However, this study also has several limitations. First, although twelve studies were included in the analysis, the number of patients was relatively small. A larger sample size is needed to obtain more reliable results. Second, some risk factors for the development and progression of gastric cancer, such as smoking, Helicobacter pylori infection, and drinking, were not considered in this study, which may affect the reliability of the results. Third, all included studies were observational in design, and selection bias should not be neglected. Fourth, the miR-200c was detected either via RT-PCR or microarray, and the use of different detection methods might affect the expression of miR-200c. Previous studies have compared the capability of microarray to quantify mRNA with that of RT-PCR and found a low correlation between these two methods [34]. Only moderate mRNA expression levels with overlap in the location of PCR primers and microarray probes can yield good agreement between these two methods [35]. Fifth, the incidence rate of gastric cancer varied between the ethnicities. Moreover, the level of miR-200c expression was different in the tissue samples compared with the blood sample. Although we conducted a subgroup analysis to detect the difference, the number of included studies was small, which would reduce the detection power. Future studies should address these limitations to accurately validate the diagnostic and prognostic value of miR-200c in gastric cancer.

## 5. Conclusion

This meta-analysis demonstrates that both serum and tissue miR-200c expression have moderate diagnostic accuracy in gastric cancer. miR-200c can be a valuable indicator for predicting the prognosis of gastric cancer. Well-designed studies with larger sample sizes are needed to further validate our results.

## Figures and Tables

**Table 1:** Characteristics of the studies that related to the diagnosis of gastric cancer.

Study	Country/year	Design	Sample type	Tumor/control	Stage	Cut-off	Test method	Sensitivity	Specificity	QUADAS
Tseng CW	China/2011	R	Tissue	22/22	I-IV	2670.5	Microarray	54.5%	54.5%	10
Valladares-Ayerbes	Spain/2012	R	Blood	52/15	I-IV	62.4	qRT-PCR	65.4%	100%	13
Lin GY	China/2013	R	Blood	50/50	I-IV	0.12	qRT-PCR	67.5%	78.5%	11
Tang JL	China/2015	R	Blood	47/50	I-IV	1.285	qRT-PCR	97.0%	54.0%	12
Keller A	Germany/2014	R	Tissue	11/93	I-IV	101.04	Microarray	72.7%	55.9%	12
Sierzega M	Poland/2017	R	Tissue	20/20	I-IV	0.342	Microarray	60.0%	45.0%	13

R: retrospective; QUADAS: quality assessment of diagnostic accuracy studies.

**Table 2:** Characteristics of the studies that related to the prognosis of gastric cancer.

Study	Country/year	Design	Sample	Number	Stage	Cut-off	Test method	Outcome	HR	95% CI	QUADAS
Valladares-Ayerbes	Spain/2012	R	Blood	52	I-IV	104.8	qRT-PCR	OS	2.24	1.091-4.614	13
								PFS	2.27	1.093-4.712	
Tang HL	China/2013	R	Tissue	126	I-IV	2.00	qRT-PCR	OS	2.29	1.38-3.81	11
								DFS	1.83	1.15-2.92	
Blanco-Calvo	Spain 2014	R	Blood	42	I-IV	Median	qRT-PCR	OS	2.24	1.091-4.614	12
								PFS	2.27	1.093-4.712	
Song FJ	China/2014	R	Blood	385	I-IV	Median	qRT-PCR	OS	1.32	0.82-2.12	12
								PFS	1.06	0.70-1.60	
Zhang HP	China/2015	R	Blood	98	I-IV	Median	qRT-PCR	OS	4.01	2.67-10.02	11
Zhou X	China/2015	R	Tissue	63	IIB-IV	Median	qRT-PCR	DFS	1.70	1.21-2.38	12
Zhang L	China/2017	R	Tissue	169	I-IV	Median	qRT-PCR	DFS	1.58	0.72–3.44	13

R: retrospective; QUADAS: quality assessment of diagnostic accuracy studies; OS: overall survival; PFS: progression-free survival; DFS: disease-free survival.

**Table 3:** Subgroup analysis of the diagnostic value of miR-200c in gastric cancer.

	Subgroup	Sensitivity	P1	Specificity	P2
Sample type	Blood	0.65 (0.41-0.88)	0.19	0.71 (0.48-0.93)	0.78
	Tissue	0.81 (0.64-0.97)		0.61 (0.38-0.83)	
Test method	qRT-PCR	0.65 (0.41-0.88)	0.19	0.71 (0.48-0.93)	0.78
	Microarray	0.81 (0.64-0.97)		0.61 (0.38-0.83)	
Ethnicity	Asian	0.80 (0.63-0.97)	0.58	0.69 (0.44-0.93)	0.99
	Caucasian	0.66 (0.42-0.90)		0.64 (0.42-0.86)	

## Data Availability

The data used to support the findings of this study are available from the corresponding author upon request.

## Abbreviations

PLR: Positive likelihood ratio
NLR: Negative likelihood ratio
DOR: Diagnostic odds ratio
AUC: Area under curve
HR: Pooled hazard ratio
CI: Confidence intervals.

## Supplementary Materials

Supplementary Materials Supplementary material: (1) search strategy of PubMed database; (2) part of the excluded studies.
