# Supplementary material for: The Diagnostic and Prognostic Value of miR-200c in Gastric Cancer: A Meta-Analysis
Source: Dis Markers. 2019 Apr 4;2019:8949618. doi: 10.1155/2019/8949618 (PMC6476052; doi:10.1155/2019/8949618)
Supplement: Supplementary Materials — Supplementary material: (1) search strategy of PubMed database; (2) part of the excluded studies. [file 8949618.f1.pdf]

Title of 40 excluded studies

1: Hwang J, Min BH, Jang J, Kang SY, Bae H, Jang SS, Kim JI, Kim KM. MicroRNA Expression Profiles in Gastric Carcinogenesis. Sci Rep. 2018 Sep 26;8(1):14393. doi: 10.1038/s41598-018-32782-8.

2: Liu S, Tian Y, Zhu C, Yang X, Sun Q. High miR-718 Suppresses Phosphatase and Tensin Homolog (PTEN) Expression and Correlates to Unfavorable Prognosis in Gastric Cancer. Med Sci Monit. 2018 Aug 22;24:5840-5850. doi: 10.12659/MSM.909527. PubMed PMID: 30131483;

3: Takei Y, Shen G, Morita-Kondo A, Hara T, Mihara K, Yanagihara K. MicroRNAs Associated with Epithelial-Mesenchymal Transition Can Be Targeted to Inhibit Peritoneal Dissemination of Human Scirrhous Gastric Cancers. Pathobiology. 2018;85(4):232-246. doi: 10.1159/000488801.

4: Ding K, Tan S, Huang X, Wang X, Li X, Fan R, Zhu Y, Lobie PE, Wang W, Wu Z. GSE1 predicts poor survival outcome in gastric cancer patients by SLC7A5 enhancement of tumor growth and metastasis. J Biol Chem. 2018 Mar 16;293(11):3949-3964. doi: 10.1074/jbc.RA117.001103.

5: Zhou X, Men X, Zhao R, Han J, Fan Z, Wang Y, Lv Y, Zuo J, Zhao L, Sang M, Liu XD, Shan B. miR-200c inhibits TGF-β-induced-EMT to restore trastuzumab sensitivity by targeting ZEB1 and ZEB2 in gastric cancer. Cancer Gene Ther. 2018 May;25(3-4):68-76. doi: 10.1038/s41417-017-0005-y.

6: Wei W, Shi L, Chen W, Hu L, Chen D, Shi X, Xiang H, Guo C, Wu Z. miR-200c regulates the proliferation, apoptosis and invasion of gastric carcinoma cells through the downregulation of EDNRA expression. Int J Mol Med. 2018 Mar;41(3):1619-1626. doi: 10.3892/ijmm.2017.3317.

7: Kurata A, Yamada M, Ohno SI, Inoue S, Hashimoto H, Fujita K, Takanashi M, Kuroda M. Expression level of microRNA-200c is associated with cell morphology in vitro and histological differentiation through regulation of ZEB1/2 and E-cadherin in gastric carcinoma. Oncol Rep. 2018 Jan;39(1):91-100. doi: 10.3892/or.2017.6093.

8: Wang Y, Zeng J, Pan J, Geng X, Liu Y, Wu J, Song P, Wang Y, Jia J, Wang L. MicroRNA-200c is involved in proliferation of gastric cancer by directly repressing

p27(Kip1). Biochem Biophys Rep. 2016 Sep 19;8:227-233. doi: 10.1016/j.bbrep.2016.09.007.
9: Li M, Gu K, Liu W, Xie X, Huang X. MicroRNA-200c as a prognostic and sensitivity marker for platinum chemotherapy in advanced gastric cancer. Oncotarget. 2017 Apr 13;8(31):51190-51199. doi: 10.18632/oncotarget.17087.
10: Jiang T, Dong P, Li L, Ma X, Xu P, Zhu H, Wang Y, Yang B, Liu K, Liu J, Xue J, Lv R, Su P, Kong G, Chang Y, Zhao C, Wang L. MicroRNA-200c regulates cisplatin resistance by targeting ZEB2 in human gastric cancer cells. Oncol Rep. 2017 Jul;38(1):151-158. doi: 10.3892/or.2017.5659.
11: Suárez-Arriaga MC, Torres J, Camorlinga-Ponce M, Gómez-Delgado A, Piña-Sánchez P, Valdez-Salazar HA, Ribas-Aparicio RM, Fuentes-Pananá EM, Ruiz-Tachiquín ME. A proposed method for the relative quantification of levels of circulating microRNAs in the plasma of gastric cancer patients. Oncol Lett. 2017 May;13(5):3109-3117. doi: 10.3892/ol.2017.5816.
12: Xu L, Zhang Y, Qu X, Che X, Guo T, Cai Y, Li A, Li D, Li C, Wen T, Fan Y, Hou K, Ma Y, Hu X, Liu Y. E3 Ubiquitin Ligase Cbl-b Prevents Tumor Metastasis by Maintaining the Epithelial Phenotype in Multiple Drug-Resistant Gastric and Breast Cancer Cells. Neoplasia. 2017 Apr;19(4):374-382. doi: 10.1016/j.neo.2017.01.011.
13: Zhang H, Sun Z, Li Y, Fan D, Jiang H. MicroRNA-200c binding to FN1 suppresses the proliferation, migration and invasion of gastric cancer cells. Biomed Pharmacother. 2017 Apr;88:285-292. doi: 10.1016/j.biopha.2017.01.023.
14: Zhang L, Huang Z, Zhang H, Zhu M, Zhu W, Zhou X, Liu P. Prognostic value of candidate microRNAs in gastric cancer: A validation study. Cancer Biomark. 2017;18(3):221-230. doi: 10.3233/CBM-160091.
15: Li Y, Nie Y, Tu S, Wang H, Zhou Y, Du Y, Cao J, Ye M. Epigenetically deregulated miR-200c is involved in a negative feedback loop with DNMT3a in gastric cancer cells. Oncol Rep. 2016 Oct;36(4):2108-16. doi: 10.3892/or.2016.4996.
16: Imaoka H, Toiyama Y, Okigami M, Yasuda H, Saigusa S, Ohi M, Tanaka K, Inoue Y, Mohri Y, Kusunoki M. Circulating microRNA-203 predicts metastases, early recurrence, and poor prognosis in human gastric cancer. Gastric Cancer. 2016 Jul;19(3):744-53. doi: 10.1007/s10120-015-0521-0.

17: Chang L, Guo F, Huo B, Lv Y, Wang Y, Liu W. Expression and clinical significance of the microRNA-200 family in gastric cancer. Oncol Lett. 2015 May;9(5):2317-2324.

18: Saito T, Kurashige J, Nambara S, Komatsu H, Hirata H, Ueda M, Sakimura S, Uchi R, Takano Y, Shinden Y, Iguchi T, Eguchi H, Ehata S, Murakami K, Sugimachi K, Mimori K. A Long Non-coding RNA Activated by Transforming Growth Factor-β is an Independent Prognostic Marker of Gastric Cancer. Ann Surg Oncol. 2015 Dec;22 Suppl 3:S915-22. doi: 10.1245/s10434-015-4554-8.

19: Zhou X, Wang Y, Shan B, Han J, Zhu H, Lv Y, Fan X, Sang M, Liu XD, Liu W. The downregulation of miR-200c/141 promotes ZEB1/2 expression and gastric cancer progression. Med Oncol. 2015 Jan;32(1):428. doi: 10.1007/s12032-014-0428-3.

20: Blanco-Calvo M, Tarrío N, Reboredo M, Haz-Conde M, García J, Quindós M, Figueroa A, Antón-Aparicio L, Calvo L, Valladares-Ayerbes M. Circulating levels of GDF15, MMP7 and miR-200c as a poor prognostic signature in gastric cancer. Future Oncol. 2014 May;10(7):1187-202. doi: 10.2217/fon.13.263.

21: Li H, Xu L, Li C, Zhao L, Ma Y, Zheng H, Li Z, Zhang Y, Wang R, Liu Y, Qu X. Ubiquitin ligase Cbl-b represses IGF-I-induced epithelial mesenchymal transition via ZEB2 and microRNA-200c regulation in gastric cancer cells. Mol Cancer. 2014 Jun 2;13:136. doi: 10.1186/1476-4598-13-136.

22: Cui FB, Liu Q, Li RT, Shen J, Wu PY, Yu LX, Hu WJ, Wu FL, Jiang CP, Yue GF, Qian XP, Jiang XQ, Liu BR. Enhancement of radiotherapy efficacy by miR-200c-loaded gelatinase-stimuli PEG-Pep-PCL nanoparticles in gastric cancer cells. Int J Nanomedicine. 2014 May 13;9:2345-58. doi: 10.2147/IJN.S60874.

23: Song F, Yang D, Liu B, Guo Y, Zheng H, Li L, Wang T, Yu J, Zhao Y, Niu R, Liang H, Winkler H, Zhang W, Hao X, Chen K. Integrated microRNA network analyses identify a poor-prognosis subtype of gastric cancer characterized by the miR-200 family. Clin Cancer Res. 2014 Feb 15;20(4):878-89. doi: 10.1158/1078-0432.CCR-13-1844.

24: Chang L, Guo F, Wang Y, Lv Y, Huo B, Wang L, Liu W. MicroRNA-200c regulates the sensitivity of chemotherapy of gastric cancer SGC7901/DDP cells by directly targeting RhoE. Pathol Oncol Res. 2014 Jan;20(1):93-8. doi: 10.1007/s12253-013-9664-7.

25: Liu Q, Li RT, Qian HQ, Wei J, Xie L, Shen J, Yang M, Qian XP, Yu LX, Jiang XQ, Liu BR. Targeted delivery of miR-200c/DOC to inhibit cancer stem cells and cancer cells by the

gelatinases-stimuli nanoparticles. Biomaterials. 2013 Sep;34(29):7191-203. doi: 10.1016/j.biomaterials.2013.06.004.

26: Li Y, Nie Y, Cao J, Tu S, Lin Y, Du Y, Li Y. G-A variant in miR-200c binding site of EFNA1 alters susceptibility to gastric cancer. Mol Carcinog. 2014 Mar;53(3):219-29. doi: 10.1002/mc.21966.

27: Zhang M, Dong BB, Lu M, Zheng MJ, Chen H, Ding JZ, Xu AM, Xu YH. miR-429 functions as a tumor suppressor by targeting FSCN1 in gastric cancer cells. Onco Targets Ther. 2016 Mar 3;9:1123-33. doi: 10.2147/OTT.S91879.

28: Imaoka H, Toiyama Y, Okigami M, Yasuda H, Saigusa S, Ohi M, Tanaka K, Inoue Y, Mohri Y, Kusunoki M. Circulating microRNA-203 predicts metastases, early recurrence, and poor prognosis in human gastric cancer. Gastric Cancer. 2016 Jul;19(3):744-53. doi: 10.1007/s10120-015-0521-0.

29: Chang L, Guo F, Huo B, Lv Y, Wang Y, Liu W. Expression and clinical significance of the microRNA-200 family in gastric cancer. Oncol Lett. 2015 May;9(5):2317-2324.

30: Ning X, Shi Z, Liu X, Zhang A, Han L, Jiang K, Kang C, Zhang Q. DNMT1 and EZH2 mediated methylation silences the microRNA-200b/a/429 gene and promotes tumor progression. Cancer Lett. 2015 Apr 10;359(2):198-205. doi: 10.1016/j.canlet.2015.01.005.

31: Zhou X, Wang Y, Shan B, Han J, Zhu H, Lv Y, Fan X, Sang M, Liu XD, Liu W. The downregulation of miR-200c/141 promotes ZEB1/2 expression and gastric cancer progression. Med Oncol. 2015 Jan;32(1):428. doi: 10.1007/s12032-014-0428-3.

32: Kurashige J, Mima K, Sawada G, Takahashi Y, Eguchi H, Sugimachi K, Mori M, Yanagihara K, Yashiro M, Hirakawa K, Baba H, Mimori K. Epigenetic modulation and repression of miR-200b by cancer-associated fibroblasts contribute to cancer invasion and peritoneal dissemination in gastric cancer. Carcinogenesis. 2015 Jan;36(1):133-41. doi: 10.1093/carcin/bgu232.

[1] 宁向红,郭蓉,韩磊,张安玲,刘茜,李朝霞,康春生,张庆瑜. DZNep 通过上调 miR-200c 表达延缓 MGC-803 胃癌细胞侵袭和迁移过程[J]. 生理学报,2015,67(01):83-89.

[2] 陈志凯,傅松维,鲍舟君,李卓栋. 胃癌患者血清 miR-200b 和 miR-200c 水平变化及临床意义[J]. 中国生化药物杂志,2016,36(05):201-203+206.

[3] 林国友,钟海兵,张雪燕,彭永剑. 血清 miR-200c 水平对胃癌的诊断价值[J]. 浙江医

学,2013,35(10):909-910+913.
[4] 周欣亮,张璁,王玉栋,赵连梅,桑梅香,单保恩. 胃癌中 DNA 甲基化对 miR-200c 141 表达影响的研究[J]. 中国肿瘤临床,2017,44(02):73-77.
[5] 周欣亮,王玉栋,张难,赵连梅,施明亮,单保恩. 胃癌组织 TGF-β 对 miR-200c/141 表达影响的研究[J]. 中华肿瘤防治杂志,2017,24(08):552-556.
[6] 周欣亮,张璁,袁虎方,王玉栋,赵连梅,桑梅香,单保恩. miR-200c 在胃癌中的表达水平与患者临床病理特征的关系[J]. 中国肿瘤生物治疗杂志,2017,24(05):538-543.
[7] 伍菲菲,谭志琴,唐云云,谢黎明,张志伟. miR-200c 对人胃癌细胞增殖能力的影响及其机制探讨[J]. 山东医药,2013,53(32):1-3.
[8] 黄俊,伍菲菲,贺利恒,张志伟. miR-200c 在胃癌组织中的表达及其临床意义[J]. 亚太传统医药,2014,10(08):103-104.

Search strategy of PubMed database

("stomach neoplasms"[MeSH Terms] OR ("stomach"[All Fields] AND "neoplasms"[All Fields]) OR "stomach neoplasms"[All Fields] OR ("gastric"[All Fields] AND "cancer"[All Fields]) OR "gastric cancer"[All Fields]) AND miR-200c[All Fields]
